# Oncological Outcome of Minimally Invasive Single-Port Segmentectomy Compared to Lobectomy for Stage IA Lung Cancer

**DOI:** 10.3390/cancers17213431

**Published:** 2025-10-25

**Authors:** Boris Kostovski, Konstantinos Gioutsos, Michail Galanis, Francine Binelli, Thanh-Long Nguyen, Patrick Dorn

**Affiliations:** Department of Thoracic Surgery, Inselspital, University Hospital of Bern, 3010 Bern, Switzerland; boris.kostovski@gmail.com (B.K.);

**Keywords:** non-small cell lung cancer, segmentectomy, lobectomy, uniportal VATS, early-stage lung cancer, stage IA3

## Abstract

**Simple Summary:**

Surgery is the primary treatment for early-stage lung cancer, and traditionally, it involves removing an entire lung lobe. In recent years, smaller and less invasive operations—such as segmentectomy, which removes only part of the lobe—have become more common. In this study, we examined the long-term outcomes of over 230 patients who underwent surgery through a minimally invasive “uniportal” technique. We found that, for tumors up to 2 cm in size, segmentectomy provided outcomes similar to lobectomy. In tumors between 2 and 3 cm, we observed an increased risk of recurrence and a lower overall survival following segmentectomy, although these differences were not significant. These findings suggest that segmentectomy is a valid oncological treatment for selected patients with specific lung cancer characteristics, preserving more lung tissue, and can be a solid alternative for these individuals. Our results may help guide surgeons in choosing the most appropriate approach based on individual tumor size and characteristics.

**Abstract:**

*Background and Objectives:* Lobectomy has traditionally been the gold standard for surgical treatment of early-stage non-small cell lung cancer (NSCLC). However, recent randomized trials suggest anatomical segmentectomy may offer comparable outcomes for selected patients with small, peripheral tumors. The role of segmentectomy in stage IA3 tumors remains less apparent in the context of video-assisted thoracoscopic surgery. *Methods:* This retrospective study analyzed 232 patients with pathological stage IA NSCLC who underwent uniportal anatomical segmentectomy (*n* = 160) or lobectomy (*n* = 72). Clinicopathological characteristics, recurrence rates, and overall survival (OS) were compared, with subgroup analysis for IA1–IA3 tumors. *Results:* The 5-year OS was 76.9% for segmentectomy and 87.5% for lobectomy (*p* = 0.105). Recurrence occurred in 15.8% of segmentectomy patients and 11.3% of lobectomy patients. In IA3 tumors, recurrence rates were higher after segmentectomy (23.5% vs. 18.2%), though not statistically significant. Lymphatic invasion was an independent predictor of mortality. No significant differences were found in tumor size, histologic subtype, or nodal involvement between groups. *Conclusions:* Uniportal anatomical segmentectomy may be a feasible alternative to lobectomy for stage IA NSCLC, especially for tumors ≤ 2 cm. For IA3 tumors, caution is advised given a trend toward worse outcomes. Careful patient selection and adherence to oncologic principles are essential.

## 1. Introduction

Since 1995, Landrenau et al. have set lobectomy as a gold standard for the surgical treatment of early-stage non-small cell lung cancer (NSCLC) compared to non-anatomical limited lung resection. Earlier studies also emphasized improved oncologic outcomes for lobectomy over wedge resection in stage I NSCLC [1], particularly following the pivotal 1995 Lung Cancer Study Group trial, which demonstrated lower local recurrence and lung cancer-specific mortality compared to limited resection [2]. With increasing rates of early detection, particularly of small, peripheral stage IA tumors, interest in less extensive surgical approaches, such as segmentectomy, has grown. Tumor size is now recognized as a key determinant in both treatment planning and prognosis, with stage IA tumors subdivided into IA1 ( ≤ 1 cm), IA2 (1–2 cm), and IA3 (2–3 cm), according to the 8th edition of the IASLC staging system [3].

Recent randomized trials have provided strong evidence in support of the use of anatomical segmentectomy in carefully selected patients. Some studies have reported comparable survival between segmentectomy and lobectomy for stage IA1 (≤ 1 cm) tumors, whereas outcomes for larger tumors remain debated [4]. The JCOG0802/WJOG4607L trial showed superior overall survival and non-inferior relapse-free survival for segmentectomy compared to lobectomy in patients with clinical stage IA tumors ≤ 2 cm [5]. Similarly, the CALGB 140,503 trial demonstrated the non-inferiority of sublobar resection (segmentectomy or wedge) compared to lobectomy in terms of disease-free and overall survival for peripheral tumors ≤ 2 cm with pathologically confirmed N0 status [6]. These findings have led to an increasing acceptance of segmentectomy beyond high-risk patients, particularly when oncologic principles—such as ensuring sufficient resection margins and performing systematic lymphadenectomy—are adhered to.

Additional factors such as spread through air spaces (STAS) have emerged as important predictors of recurrence after limited resection [7]. A post hoc analysis of the JCOG0802/WJOG4607L randomized trial by Hattori et al. demonstrated distinct recurrence patterns between segmentectomy and lobectomy in patients with small-sized, radiologically solid NSCLC, emphasizing the importance of careful patient selection when considering sublobar resection [8]. Concurrently, the development and refinement of minimally invasive surgical techniques—most notably, uniportal video-assisted thoracoscopic surgery (uVATS)—have enhanced the technical feasibility of segmentectomy while reducing perioperative morbidity [9,10,11]. These advances have further expanded the role of segmentectomy in modern thoracic surgical practice, allowing it to be considered in standard-risk populations. National registry data from the National Cancer Database (NCDB) indicate that the proportion of segmentectomies for stage IA NSCLC nearly doubled, increasing from 3.3% in 2004 to 6.1% in 2018 [12].

A major area of clinical debate remains the role of segmentectomy in stage IA3 NSCLC (tumors 2.1–3.0 cm). A 2023 meta-analysis by Zhang et al. concluded that lobectomy was associated with improved overall and disease-free survival compared to segmentectomy for tumors in this size range [13]. Conversely, a recent multicenter European study using inverse probability of treatment weighting (IPTW) showed no significant difference in recurrence-free survival between stage IA3 and earlier stages when treated with segmentectomy, suggesting feasibility in carefully selected patients [9].

In addition to tumor size, histologic features such as spread through air spaces (STAS) have emerged as relevant prognostic markers. STAS is associated with increased risk of recurrence and has been considered a relative contraindication for sublobar resection since its introduction. However, recent data suggest that segmentectomy offers comparable outcomes to lobectomy in STAS-positive tumors, provided that resection margins and lymphadenectomy are adequate [14].

Current clinical guidelines have evolved to reflect these data. The National Comprehensive Cancer Network (NCCN) now recognizes segmentectomy as an acceptable alternative to lobectomy for peripheral tumors ≤ 2 cm in diameter without nodal involvement, particularly in patients with limited cardiopulmonary reserve or other factors that increase surgical risk [15]. Similarly, the American College of Chest Physicians (ACCP) recommends segmentectomy in patients with limited cardiopulmonary reserve or those with small peripheral tumors where lobectomy may be excessive [16].

Given this rapidly shifting landscape, further evaluation of segmentectomy in broader stage IA subgroups, particularly IA3, is warranted. The present study aims to contribute to this discussion by comparing long-term outcomes between uniportal anatomical segmentectomy and lobectomy for stage IA NSCLC, and identifying predictors of recurrence and survival based on histopathologic and clinical variables.

## 2. Materials and Methods

This was a retrospective, single-center cohort study conducted at the Department of Thoracic Surgery, Inselspital, Bern University Hospital, University of Bern, Switzerland.

All patients who underwent uniportal video-assisted thoracoscopic surgery (uVATS) for pathological stage IA non-small cell lung cancer (NSCLC) between 1 January 2015, and 31 December 2021, were included in this retrospective study period and were identified through the hospital’s electronic medical records system.

Surgical procedures included either anatomical segmentectomy or lobectomy performed using the uniportal VATS approach. Segmentectomy involved the anatomical resection of one pulmonary segment, including systematic dissection and division of the segmental artery, segmental bronchus, intersegmental vein, and precise separation along the anatomical intersegmental planes, while lobectomy entailed the removal of an entire pulmonary lobe. Systematic mediastinal and hilar lymph node dissection was performed in all cases in accordance with the guidelines of the European Society of Thoracic Surgeons (ESTS) [17]. For right upper and middle lobe resections (including segmentectomies originating from these lobes), mediastinal stations 2R, 4R, and 7 were routinely dissected; for right lower lobe resections, stations 4R, 7, 8, and 9 were included. For left upper lobe resections, stations 5, 6, and 7 were dissected, whereas for left lower lobe resections, stations 7, 8, and 9 were included. In all cases, ipsilateral hilar (10), interlobar (11), and, when applicable, segmental or subsegmental nodes (stations 12 and 13) were also removed during segmentectomy procedures.

Clinical and oncologic data collected included age, sex, tumor size and location, histologic subtype and grading, lymphovascular and pleural invasion, radiological distance to pleura, number of resected lymph nodes and nodal stations, pathologic stage, date of surgery, 5-year overall survival, and recurrence pattern (locoregional or distant). Recurrence sites were categorized as local (same lobe or staple line), regional (ipsilateral hilar or mediastinal lymph nodes), or distant (contralateral hilar or mediastinal lymph nodes, contralateral lung, or extrathoracic organ metastases).

Postoperative follow-up was conducted according to our institutional protocol, with computed tomography (CT) imaging every six months for the first two years and annually thereafter for five years in total.

The primary endpoint was 5-year overall survival. The secondary endpoint was tumor recurrence. Radiologic distance to the pleura, tumor diameter, and the number of harvested lymph nodes and nodal stations were evaluated as potential prognostic factors.

Statistical analysis was conducted using STATA version 16. Overall survival was estimated using the Kaplan–Meier method and compared using the log-rank test. A Cox proportional hazards model was used to determine hazard ratios (HR) and their 95% confidence intervals. Segmentectomy was considered non-inferior to lobectomy if the upper bound of the one-sided 95% confidence interval for the HR did not exceed 1.306. Univariate and multivariate logistic regression analyses were used to identify predictors for mortality and recurrence. A *p*-value ≤ 0.05 was considered statistically significant.

## 3. Results

### 3.1. Patient Cohort and Baseline Characteristics

Table 1 and Table 2 summarize the baseline demographic and clinicopathological characteristics of the study population stratified by type of operation. A total of 232 patients with pathological stage IA non-small cell lung cancer (NSCLC) were included in the analysis. Of these, 160 patients underwent uniportal anatomical segmentectomy and 72 patients underwent uniportal lobectomy.

Baseline characteristics, including age, gender distribution, tumor size, histologic subtype, and tumor stage, showed no statistically significant differences between the segmentectomy and lobectomy groups (*p* > 0.05 for all comparisons).

The mean age of the patients was 67.3 ± 8.7 years in the segmentectomy group and 65.4 ± 11.2 years in the lobectomy group. The gender distribution was similar, with males comprising 54.4% of the segmentectomy group and 54.2% of the lobectomy group.

The mean tumor size was 1.6 ± 0.6 cm in the segmentectomy group and 1.7 ± 0.6 cm in the lobectomy group. Adenocarcinoma was the predominant histologic subtype in both groups, comprising 68.8% of tumors in the segmentectomy group and 65.3% in the lobectomy group.

Tumor stages IA1, IA2, and IA3 were distributed without significant differences between the segmentectomy and lobectomy groups (*p* > 0.05).

Lymphatic invasion (L) was observed in 7.0% of patients in the segmentectomy group and 14.5% in the lobectomy group, while vascular invasion (V) was found in 18.5% and 23.2% of cases, respectively. R0 resection was achieved in over 98% of cases. In total, R0 resection was not achieved in three patients. Two patients in the segmentectomy group had insufficient margins, while one patient in the lobectomy group had an R1 situation. The patient with R1 resection received adjuvant radiochemotherapy. The two segmentectomy patients were managed with close follow-up, without additional therapy.

The mean radiological distance from the tumor to the pleura was 11.8 ± 11.6 mm in the segmentectomy group and 14 ± 16.2 mm in the lobectomy group.

The mean number of resected lymph nodes was significantly higher in the lobectomy group (14.1 ± 8.7) than in the segmentectomy group (10.4 ± 7.4).

The number of sampled lymph node stations was 4.7 ± 2 for segmentectomy and 5 ± 1.6 for lobectomy.

### 3.2. Overall Survival

**Figure 1:**
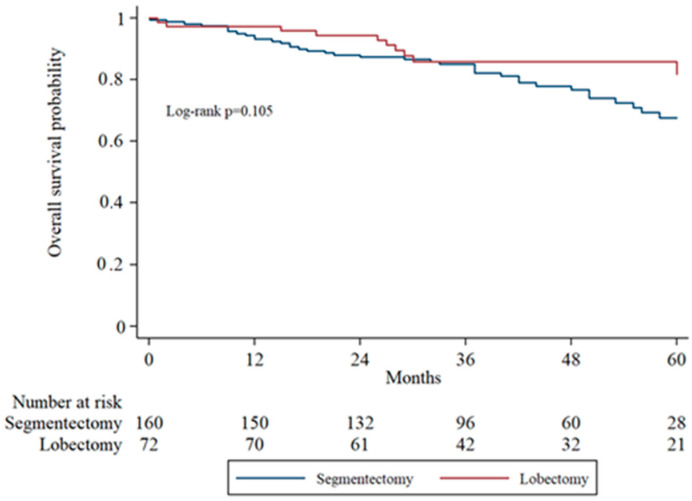
Kaplan–Meier overall survival analysis comparing segmentectomy and lobectomy groups in the entire cohort.

Overall survival (OS) was assessed using Kaplan–Meier analysis and compared between groups using the log-rank test. The 5-year overall survival was 76.9% in the segmentectomy group and 87.5% in the lobectomy group. Although the lobectomy group demonstrated a trend toward improved survival, the difference was not statistically significant (log-rank *p* = 0.105). Figure 1 illustrates the survival curves for the two surgical approaches.

### 3.3. Subgroup Analysis of Overall Survival by Tumor Stage

**Figure 2:**
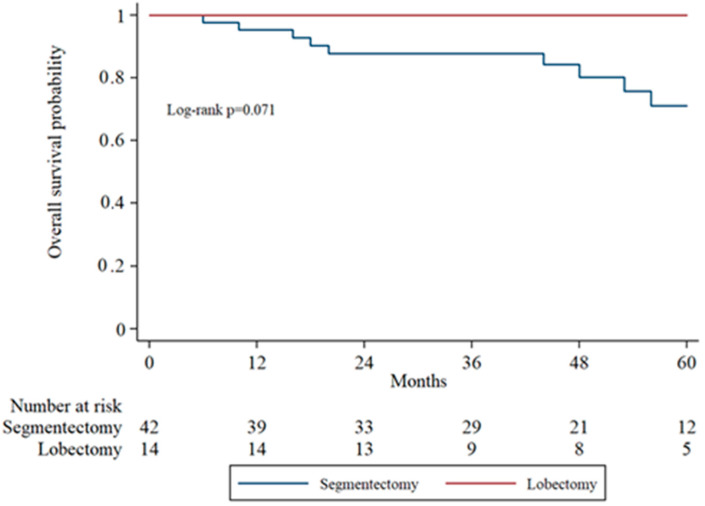
Kaplan–Meier overall survival analysis in IA1 patients comparing segmentectomy and lobectomy.

**Figure 3:**
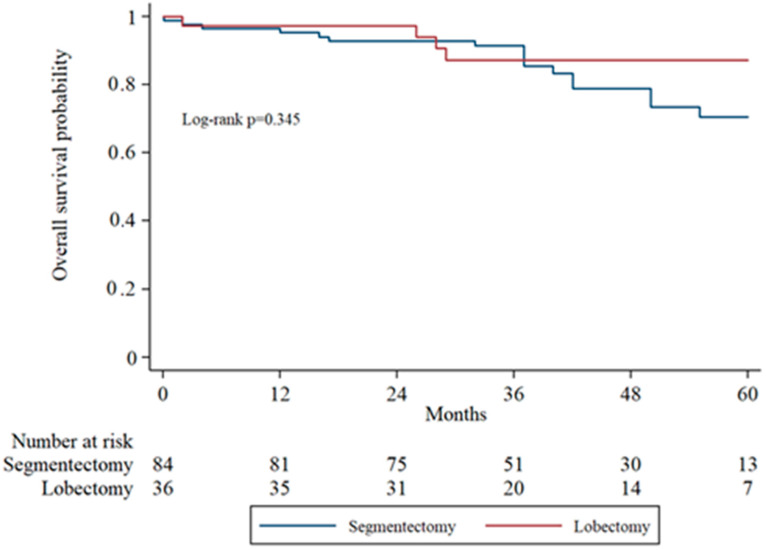
Kaplan–Meier overall survival analysis in IA2 patients comparing segmentectomy and lobectomy.

**Figure 4:**
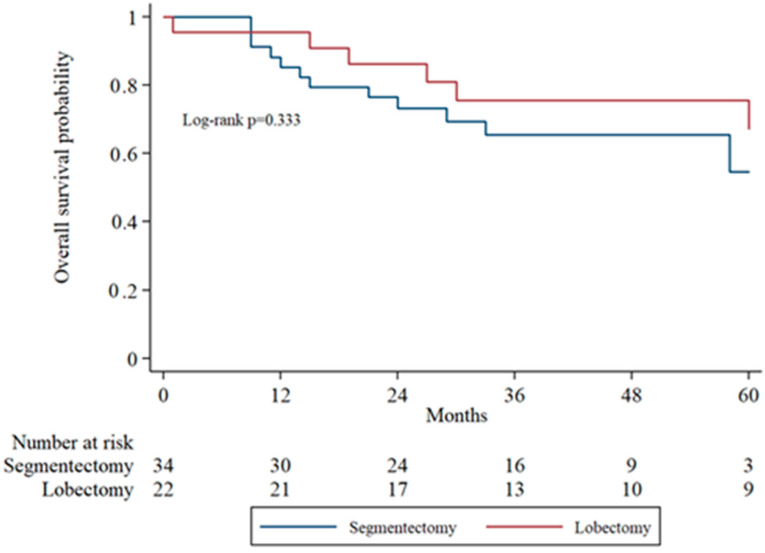
Kaplan–Meier overall survival analysis in IA3 patients comparing segmentectomy and lobectomy.

Subgroup analysis of overall survival (OS) by tumor stage (IA1, IA2, IA3) was performed to evaluate surgical outcomes across increasing tumor sizes. In IA1 patients, the 5-year OS was higher in the lobectomy group compared to the segmentectomy group, though the difference was not statistically significant (log-rank *p* = 0.071). In IA2 patients, OS remained comparable between groups without significant divergence in survival curves. In the IA3 subgroup, there was no statistically significant difference in overall survival between the segmentectomy and lobectomy groups (*p* = 0.333). Figure 2, Figure 3 and Figure 4 display the Kaplan–Meier survival curves stratified by stage and type of operation.

### 3.4. Recurrence

Cancer recurrence was observed in 8 of 72 patients (11.1%, 95% CI 5.7–20.4%) after lobectomy and in 24 of 160 patients (15.0%, 95% CI 10.3–21.3%) after segmentectomy. The difference was not statistically significant (odds ratio 0.71, 95% CI 0.30–1.66; *p* = 0.54).

A regression analysis was performed to identify predictors of recurrence, as shown in Table 3. Subgroup analysis by tumor stage revealed a trend toward higher recurrence following segmentectomy across all IA subgroups: 7.1% vs. 0% for IA1, 18.0% vs. 13.0% for IA2, and 23.5% vs. 18.2% for IA3 (segmentectomy vs. lobectomy, respectively). Although these differences were not statistically significant (*p* > 0.05 for all comparisons), a trend toward higher recurrence rates was observed in patients with stage IA3 tumors compared to those with IA1 and IA2 tumors, regardless of the type of surgery.

### 3.5. Prognostic Factors for Overall Survival

Table 4 summarizes the results of univariate and multivariate logistic regression analyses evaluating predictors of overall survival. In univariate analysis, male gender, increasing age, squamous histology, and lymphatic invasion (L1) were found to be significantly associated with worse overall survival. In the multivariate model, only male gender (OR 2.99, 95% CI 1.29–6.93, *p* = 0.011) and lymphatic invasion (OR 3.51, 95% CI 1.04–11.86, *p* = 0.043) remained statistically significant. Tumor size and number of harvested lymph nodes showed trends toward significance in univariate analysis but were not independently associated with overall survival in the multivariate model.

### 3.6. Prognostic Factors for Tumor Recurrence

Table 3 summarizes the results of univariate and multivariate logistic regression analyses evaluating predictors of tumor recurrence. In the univariate analysis, vascular invasion (V1) showed a trend toward association with recurrence (OR 2.10, 95% CI 0.91–4.83, *p* = 0.082), while upper and lower lobe location appeared to confer increased odds, though not significantly. In the multivariate model, the number of nodal stations sampled was significantly associated with recurrence (OR 1.39, 95% CI 1.01–1.92, *p* = 0.049). No other variables, including surgical technique, tumor size, or pleural distance, demonstrated statistically significant associations.

### 3.7. Tumor Recurrence Based on Lobe and Segment

Cancer recurrence rates following lobectomy varied by lobe: 20.0% for the right lower lobe (RLL; 3/15, 95% CI 7.0–45.2%), 10.5% for the right upper lobe (RUL; 2/19, 95% CI 2.9–31.4%), 4.8% for the right middle lobe (RML; 1/21, 95% CI 0.9–22.7%), 10.0% for the left upper lobe (LUL; 1/10, 95% CI 1.8–40.4%), and 14.3% for the left lower lobe (LLL; 1/7, 95% CI 2.6–51.3%), as shown in Table 5. The overall comparison showed no statistically significant differences among lobectomy types. When comparing individual lobectomy types, recurrence occurred in 20.0% of patients after RLL (3/15) and 4.8% after RML (1/21). Although the observed difference suggested a lower recurrence rate after RML (odds ratio 0.20, 95% CI 0.02–2.15), this did not reach statistical significance (*p* = 0.15).

Moreover, recurrence rates varied across the different segmentectomies, with a total of 24 recurrences among 160 patients, corresponding to an overall recurrence rate of 15%. Several procedures, including resection of the left upper division, the anterior segment of the right upper lobe, and basilar segments of the right lower lobe, tended to show higher recurrence rates (Table 6 and Table 7).

### 3.8. Tumor Recurrence After Complex Versus Simple Segmentectomy by Lobe

Tumor recurrence following segmentectomy showed marked variation depending on both the anatomical location and the technical complexity of the procedure (Table 8). Segmentectomies were classified as either simple or complex according to the study published by Handa et al. [18], and recurrence patterns were analyzed accordingly.

In the right lower lobe, recurrence rates differed substantially between simple and complex procedures. Among the 18 patients who underwent simple segmentectomy, 2 recurrences were observed (11.1%). In contrast, among the 20 patients who underwent complex segmentectomy, 5 experienced recurrence (25.0%). This represents a more than twofold increase in recurrence after complex procedures in the right lower lobe.

The right upper lobe has no simple segmentectomies per se; all 44 patients underwent complex resections, of whom 7 (15.9%) developed tumor recurrence. In contrast, the left upper lobe showed a reverse pattern. Among 13 simple segmentectomies, 4 patients developed recurrence (30.8%), compared to 4 recurrences among 23 complex resections (17.4%). Notably, the simple left upper lobe segmentectomies demonstrated the highest recurrence rate of any subgroup in this analysis, exceeding even complex procedures in the same lobe. The left lower lobe demonstrated the most favorable outcomes across both complexity groups. Both simple and complex segmentectomies (20 patients each) had a single recurrence, resulting in an identical recurrence rate of 5.0%.

### 3.9. Tumor Recurrence by Lobe: Segmentectomy vs. Lobectomy

Recurrence rates varied notably between segmentectomy and lobectomy, depending on the resected lobe. In the right lower lobe, recurrence following segmentectomy was 18.4%, compared to 20.0% after lobectomy. The right upper lobe showed a slight difference, with a recurrence rate of 15.9% after segmentectomy versus 10.5% after lobectomy. A more pronounced discrepancy was observed in the left upper, where 22.2% of patients experienced recurrence after segmentectomy, compared to only 10.0% after lobectomy. In contrast, the left lower lobe demonstrated the most favorable results for segmentectomy, with a recurrence rate of 5.0%, while lobectomy in the same lobe was associated with a higher recurrence rate of 14.3%.

A chi-square approximation comparing observed versus expected recurrences across all procedures yielded a χ^2^ statistic of 32.6 with 27 degrees of freedom (*p* ≈ 0.23), indicating that overall differences among procedures were not statistically significant. Small sample sizes limited the analysis in several categories (*n* = 1–2), which may have reduced the ability to detect significant differences. 

In the segmentectomy group, which included 160 patients, there were 24 documented recurrences: 13 were locoregional, and 11 were distant. In comparison, the lobectomy group, consisting of 72 patients, experienced a total of 8 recurrences, with 3 classified as locoregional and 5 as distant. The ratio of distant to locoregional recurrences was relatively similar between the two groups. However, as with other findings related to recurrences, the interpretation of these patterns is limited due to the relatively small number of events in the lobectomy group.

## 4. Discussion

Our results contribute to the growing body of evidence supporting the role of anatomical segmentectomy as a valid alternative to lobectomy for patients with early-stage non-small cell lung cancer (NSCLC). Similar outcomes have previously been reported by Gioutsos et al., who demonstrated a 3-year overall survival of 87.9% after uniportal anatomical segmentectomy for stage IA NSCLC in a Swiss single-center cohort [19]. The 5-year overall survival observed in our cohort (76.9% for segmentectomy vs. 87.5% for lobectomy) is comparable to findings from the CALGB 140,503 and JCOG0802/WJOG4607L trials, which demonstrated non-inferior oncologic outcomes for sublobar resection in peripheral NSCLC ≤ 2 cm [5,6]. Notably, our study also included patients with stage IA3 tumors, a group typically underrepresented in prior randomized trials. Several retrospective and pooled studies have further supported the use of segmentectomy in small peripheral tumors and invasive adenocarcinoma, reporting oncologic outcomes comparable to lobectomy [20,21,22].

A trend indicating improved survival was observed in patients with IA1 tumors undergoing lobectomy (log-rank *p* = 0.071), which is somewhat unexpected. For tumors of this small size, segmentectomy is typically expected to provide comparable oncologic outcomes while preserving lung function. We lack a definitive explanation for this finding, and it may be due to the relatively small number of patients in the lobectomy subgroup. Importantly, the difference did not reach statistical significance, highlighting that both surgical approaches remain reasonable options for IA1 tumors. These results support individualized surgical decision-making, taking into account tumor characteristics, patient comorbidities, and functional reserve. Improved survival, although not statistically significant, was also observed in the IA3 subgroup. This observation suggests that tumor size ≥2 cm may still be a relevant factor when selecting patients for sublobar resection. This aligns with previous retrospective analyses suggesting reduced disease-free survival in patients undergoing segmentectomy with IA3 tumors [23]. Conversely, a recent European IPTW-based multicenter study by Huang et al. reported no significant difference in recurrence-free survival between IA3 and smaller tumors, supporting the notion that segmentectomy may still be oncologically sound in larger tumors when margins and nodal clearance are adequate [9]. The growing role of segmentectomy in stage IA NSCLC has also been supported by large-scale observational studies [24]. Prospective comparisons have similarly found segmentectomy to be oncologically acceptable in selected cases of early-stage disease [6].

The recurrence rate in our study was higher in the segmentectomy group across all IA substages, consistent with prior reports indicating an elevated risk of local recurrence after limited resection [25,26,27]. This trend may be influenced by multiple tumor-related and technical factors, which have been identified as predictors of local recurrence after sublobar resection [28]. However, the absolute difference remained modest and not statistically significant. These findings suggest that anatomical segmentectomy may be an acceptable alternative to lobectomy in carefully selected patients. In particular, segmentectomy has demonstrated favorable oncologic outcomes in ground-glass opacity (GGO)-dominant lesions, where sublobar resection may be sufficient [29]. While initial data were derived from selected subgroups such as GGO-dominant lesions, subsequent large-scale registry studies have confirmed comparable survival outcomes for segmentectomy in broader stage I NSCLC populations [30,31]. In addition to oncologic outcomes, segmentectomy has also been associated with better preservation of pulmonary function in selected patients, without compromising long-term survival [32]. However, caution is warranted, particularly for tumors ≥2 cm, where a trend toward worse outcomes was observed despite adherence to oncologic principles—including adequate margins and systemic lymphadenectomy. The length of the resection margin has also been shown to influence recurrence risk in sublobar resections directly [33]. This observation is supported by studies demonstrating that insufficient margin distance in sublobar resections is associated with higher recurrence rates [34]. Radiologic features such as the tumor-to-pleura distance have also been shown to impact the adequacy of resection margins [35].

Balancing oncologic control with preservation of lung function remains a critical consideration in surgical decision-making, particularly in sublobar resections [8,36]. While lobectomy has long been the standard, the functional benefits of segmentectomy are increasingly recognized, especially in patients with limited pulmonary function. These considerations are important when evaluating the trade-offs between recurrence risk and postoperative quality of life.

Our multivariate analysis identified lymphatic invasion as an independent predictor of mortality, highlighting the critical importance of complete lymph node assessment during segmentectomy. This supports prior findings that emphasized the prognostic value of adequate lymph node sampling in sublobar resections [37]. Other studies have also highlighted imaging-based biomarkers such as SUVmax as significant prognostic indicators in stage IA tumors [38]. Although we did not specifically evaluate spread through air spaces (STAS), this factor has been consistently associated with poorer outcomes following sublobar resections [7,39]. Certain histologic subtypes, particularly those associated with STAS, may present a higher risk of recurrence even after anatomical segmentectomy [40]. The absence of STAS data in our cohort limits direct comparison but underscores the need for thorough pathological evaluation to guide resection decisions.

All procedures in our study were performed using a uniportal video-assisted thoracoscopic approach. Comparative analyses have shown uniportal VATS to achieve similar or improved outcomes compared to multiportal techniques [41]. While this technique was not evaluated as a variable, the consistent use of a minimally invasive strategy may support the generalizability of our findings to current surgical practice.

Our findings also indicate that tumor recurrence following anatomical lung resection is influenced not only by the surgical method (segmentectomy vs. lobectomy) but also by the anatomical lobe in which the tumor is located. While recurrence rates in the RLL were comparable between segmentectomy (18.4%) and lobectomy (20.0%), other lobes showed more significant divergence.

Interestingly, our findings showed particularly poor recurrence rates in left S1–3 segmentectomies, while S6 segmentectomies bilaterally and left lower lobe procedures demonstrated very low recurrence. These results, however, conflict with previous findings [42], which described an inverse pattern. The LUL may present unique challenges due to its complex segmental anatomy and lymphatic drainage, making complete resection with adequate margins more difficult, especially during simple segmentectomy. This is reflected in our data, where simple LUL segmentectomies had the highest recurrence rate (30.8%) among all subgroups, exceeding even complex resections in the same lobe.

Conversely, the LLL appears to be a favorable site for segmentectomy. Both simple and complex procedures yielded a recurrence rate of 5.0%, supporting its potential for safe sublobar resection when oncologic criteria are met. Prior studies have similarly demonstrated satisfactory oncologic outcomes for segmentectomy in appropriately selected lower lobe tumors.

Although our overall comparison of recurrence rates between surgical subgroups did not reach statistical significance (χ^2^ = 32.6, df = 27, *p* ≈ 0.23), this may be due to limited statistical power, given the small sample sizes in several categories. The observed lobar trends, however, underscore the importance of considering lobe-specific anatomy and technical feasibility when selecting surgical approaches.

Future studies with larger, multicenter datasets are needed to confirm these findings and to refine segmentectomy indications based not only on tumor size and location, but also on individual lobe characteristics.

Several limitations must be acknowledged. The retrospective design introduces inherent selection bias, and the lack of randomized patient allocation limits causal inference. A further limitation of this study is the lack of detailed quantitative data on pathological resection margins, which prevented further analysis of margin distance in relation to tumor recurrence. Additionally, STAS data were not routinely available, and follow-up duration varied between patients. Nonetheless, the consistent application of surgical technique, comprehensive perioperative data, and inclusion of a real-world patient population strengthen the external validity of our findings. These results are in agreement with updated meta-analyses, which demonstrate similar survival rates between segmentectomy and lobectomy in selected patients [43].

## 5. Conclusions

Anatomical segmentectomy via a uniportal thoracoscopic approach may serve as an oncologically acceptable alternative to lobectomy in patients with pathological stage IA NSCLC, particularly for tumors ≤ 2 cm. Our findings demonstrated comparable overall survival and recurrence rates between segmentectomy and lobectomy across stage IA subgroups, although a trend toward higher recurrence and lower survival was observed in IA3 tumors following segmentectomy.

However, recurrence rates varied by tumor location and procedural complexity. These findings underscore the importance of individualized surgical planning, which should be based not only on tumor size but also on the anatomical lobe and technical feasibility. While segmentectomy remains a valid option for many early-stage tumors, caution is advised for IA3 lesions and for anatomically challenging resections where achieving sufficient margins and nodal assessment may be compromised. Further prospective studies are needed to refine selection criteria and define the oncologic boundaries of segmentectomy in stage IA NSCLC.

## Figures and Tables

**Table 1:** Demographic and surgical characteristics.

	Type of Operation		
	Segmentectomy	Lobectomy	*p*-Value
**Gender**			
Female	73 (45.6%)	33 (45.8%)	1
**Age**	67.3 (8.7)	65.4 (11.2)	0.1966
**Side**			
Right	84 (52.5%)	55 (76.4%)	**0.001**
**Lobe**			
Lower	77 (48.1%)	23 (31.9%)	**0.031**
Upper	83 (51.9%)	29 (40.3%)	0.135
Middle	0 (0%)	20 (27.8%)	
**Number of Segments removed**			
Single	110 (68.8%)	-	
Multiple	50 (31.2%)	-	
**Diameter (cm)**	1.59 (0.63)	1.71 (0.61)	0.172
**Lymph Node Stations**	4.66 (1.99)	5.04 (1.58)	0.121
**Lymph Nodes**	10.38 (7.43)	14.07 (8.69)	**0.002**

**Table 2:** Histological and oncological characteristics.

	Type of Operation		
	Segmentectomy	Lobectomy	*p*-Value
**Postoperative Histology**			
Adenocarcinoma	112 (70%)	48 (66.7%)	0.71
Squamous	34 (21.3%)	14 (19.4%)	0.89
Typical Carcinoid	9 (5.6%)	9 (12.5%)	0.11
Large Cell Carcinoma	4 (2.5%)	0 (0%)	0.31
Adenosquamous	1 (0.6%)	1 (1.4%)	0.53
**L (Lymphatic invasion)**			
Yes	11 (7%)	10 (14.5%)	0.14
**V (Vascular Invasion)**			
Yes	29 (18.5%)	16 (23.2%)	0.58
**Pn (Perineural Invasion)**			
Yes	3 (1.9%)	2 (2.9%)	1
**Radiological Distance to Pleura (mm)**	11.78 (11.6)	13.95 (16.18)	0.307
**Pathological Distance to Pleura (mm)**	-	0.36 (0.44)	
**Stage**			
IA1	42 (26.3%)	14 (19.4%)	0.34
IA2	84 (52.5%)	36 (50%)	0.833
IA3	34 (21.3%)	22 (30.6%)	0.172

**Table 3:** Univariate and multivariate logistic regression analyses of factors associated with tumor recurrence.

	Univariate Analysis			Multivariate Analysis		
	OR	95% CI	*p*	OR	95% CI	*p*
Type of operation, Segmentectomy	1.48	(0.63–3.47)	0.366	2.17	(0.68–6.93)	0.191
Gender, Male	1.00	(0.48–2.09)	0.996	0.75	(0.29–1.92)	0.551
Age	0.99	(0.95–1.03)	0.522	0.97	(0.92–1.02)	0.218
Right Side	1.04	(0.49–2.21)	0.921	1.29	(0.48–3.48)	0.615
Lower Lobe	2.87	(0.35–23.31)	0.323	-	-	-
Upper Lobe	3.97	(0.50–31.46)	0.192	-	-	-
Diameter (cm)	0.88	(0.48–1.60)	0.676	0.90	(0.40–2.04)	0.809
Lymph Node Stations	1.00	(0.82–1.22)	0.983	1.39	(1.01–1.92)	**0.049**
Lymph Nodes	0.97	(0.92–1.02)	0.257	0.92	(0.83–1.01)	0.086
Postoperative Histology, Squamous	1.12	(0.47–2.69)	0.800	-	-	-
Postoperative Histology, Neuroendocrine	0.00	(0–0)	0.998	-	-	-
Postoperative Histology, Other	0.55	(0.07–4.46)	0.572	-	-	-
L (Lymphatic invasion)	1.56	(0.49–5.01)	0.453	1.74	(0.44–6.96)	0.433
V (Vascular Invasion)	2.10	(0.91–4.83)	0.082	2.79	(0.97–8.02)	0.057
Grading, Grade 1	0.14	(0.02–1.12)	0.064	0.16	(0.02–1.46)	0.104
Grading, Grade 2	0.63	(0.27–1.49)	0.294	0.52	(0.19–1.39)	0.193
Radiological Distance to Pleura (mm)	0.99	(0.96–1.02)	0.444	1.00	(0.96–1.04)	0.870

**Table 4:** Univariate and multivariate logistic regression analyses of factors associated with overall survival.

	Univariate Analysis			Multivariate Analysis		
	OR	95% CI	*p*	OR	95% CI	*p*
Type of operation, Segmentectomy	1.61	(0.79–3.30)	0.192	2.09	(0.72–6.08)	0.176
Gender, Male	2.43	(1.24–4.74)	**0.009**	2.99	(1.29–6.93)	**0.011**
Age	1.04	(1.00–1.08)	**0.032**	1.04	(0.99–1.09)	0.089
Right Side	0.69	(0.37–1.30)	0.251	0.58	(0.25–1.35)	0.208
Lower Lobe	1.51	(0.40–5.63)	0.542	0.34	(0.05–2.20)	0.258
Upper Lobe	1.80	(0.49–6.61)	0.376	0.48	(0.08–2.91)	0.426
Diameter (cm)	1.38	(0.84–2.28)	0.205	1.02	(0.53–1.96)	0.962
Lymph node Stations	0.97	(0.82–1.15)	0.748	1.16	(0.88–1.51)	0.290
Lymph Nodes	0.96	(0.92–1.01)	0.112	0.97	(0.90–1.03)	0.316
Postoperative Histology, Squamous	2.14	(1.05–4.35)	**0.035**	1.85	(0.76–4.48)	0.174
Postoperative Histology, Neuroendocrine	0.26	(0.03–2.04)	0.201	1.47	(0.11–19.57)	0.768
Postoperative Histology, Other	0.39	(0.05–3.16)	0.378	0.32	(0.02–5.39)	0.432
L (Lymphatic invasion)	2.91	(1.15–7.36)	**0.024**	3.51	(1.04–11.86)	**0.043**
V (Vascular Invasion)	2.01	(0.98–4.13)	0.056	1.62	(0.64–4.10)	0.311
Grading, Grade 1	0.36	(0.11–1.19)	0.093	0.47	(0.10–2.24)	0.341
Grading, Grade 2	0.71	(0.34–1.45)	0.344	0.62	(0.26–1.49)	0.284
Radiological Distance to Pleura (mm)	0.99	(0.97–1.02)	0.917	1.00	(0.97–1.03)	0.986

**Table 5:** Recurrence by type of lobectomy.

Procedure	Performed	Recurrence	Percentage
RUL	19	2	10.5%
RML	21	1	4.8%
RLL	15	3	20%
LUL	10	1	10%
LLL	7	1	14.3%

RUL: right upper lobe; RML: right middle lobe; RLL: right lower lobe; LUL: left upper lobe; LLL: left lower lobe.

**Table 6:** Complex segmentectomies.

Procedure	Performed	Recurrence	Percentage
S1-2 L	13	3	23.1%
S1-2 R	9	1	11.1%
S1 L	1	0	0.0%
S1 R	16	1	6.3%
S10 L	6	1	16.7%
S10 R	2	0	0.0%
S2+9 R	1	0	0.0%
S2 L	5	0	0.0%
S2 R	12	1	8.3%
S3 L	3	1	33.3%
S3 R	7	4	57.1%
S5 L	1	0	0.0%
S6+10 R	1	0	0.0%
S7+10 R	1	0	0.0%
S7-10 R	2	0	0.0%
S8-10 R	2	2	100.0%
S8+9 R	1	0	0.0%
S8 L	10	0	0.0%
S8 R	6	3	50.0%
S9-10 L	2	0	0.0%
S9-10 R	4	0	0.0%
S9 L	2	0	0.0%
Total	108	17	15.7%

L: left; R: right.

**Table 7:** Simple segmentectomies.

Procedure	Performed	Recurrence	Percentage
S1-3 L	11	4	36.4%
S6 L	20	1	5.0%
S6 R	18	2	11.1%
Lingulectomy (S4-5 L)	2	0	0.0%
Total	52	7	13.5%

L: left; R: right.

**Table 8:** Recurrence by segmentectomies based on lobe and complexity.

		Performed	Recurrence	%
RUL	Simple	-	-	-
	Complex	44	7	15.9%
	Total	44	7	15.9%
RLL	Simple	18	2	11.1%
	Complex	20	5	25%
	Total	38	7	18.4%
LUL	Simple	13	4	30.8%
	Complex	23	4	17.4%
	Total	36	8	22.2%
LLL	Simple	20	1	5%
	Complex	20	1	5%
	Total	40	2	5%

RUL: right upper lobe; RLL: right lower lobe; LUL: left upper lobe; LLL: left lower lobe.

## Data Availability

The data presented in this study are available upon reasonable request from the corresponding author. The data are not publicly available.
